# Process optimisation for production and recovery of succinic acid using xylose-rich hydrolysates by *Actinobacillus succinogenes*

**DOI:** 10.1016/j.biortech.2021.126224

**Published:** 2022-01

**Authors:** Esther Oreoluwa Jokodola, Vivek Narisetty, Eulogio Castro, Sumit Durgapal, Frederic Coulon, Raveendran Sindhu, Parameswaran Binod, J. Rajesh Banu, Gopalakrishnan Kumar, Vinod Kumar

**Affiliations:** a School of Water, Energy and Environment, Cranfield University, Cranfield MK43 0AL, UK; b Department of Chemical, Environmental and Materials Engineering, Universidad de Jaén, Campus Las Lagunillas, 23071 Jaén, Spain; c Department of Pharmaceutical Sciences, Kumaun University, Bhimtal, Nainital 263136, Uttarakhand, India; d Microbial Processes and Technology Division, CSIR-National Institute for Interdisciplinary Science and Technology (CSIR-NIIST), Thiruvananthapuram 695 019, Kerala, India; e Department of Life Sciences, Central University of Tamil Nadu, Neelakudi, Thiruvarur, Tamil Nadu 610005, India; f School of Civil and Environmental Engineering, Yonsei University, Seoul 03722, Republic of Korea

**Keywords:** Succinic acid, *Actinobacillus succinogenes*, Olive pits, Sugarcane bagasse, Pure xylose, Downstream processing

## Abstract

Succinic acid (SA) is a top platform chemical obtainable from biomass. The current study evaluated the potential of *Actinobacillus succinogenes* for SA production using xylose-rich hemicellulosic fractions of two important lignocellulosic feedstocks, olive pits (OP) and sugarcane bagasse (SCB) and the results were compared with pure xylose. Initial experiments were conducted in shake flask followed by batch and fed-batch cultivation in bioreactor. Further separation of SA from the fermented broth was carried out by adapting direct crystallisation method. During fed-batch culture, maximum SA titers of 36.7, 33.6, and 28.7 g/L was achieved on pure xylose, OP and SCB hydrolysates, respectively, with same conversion yield of 0.27 g/g. The recovery yield of SA accumulated on pure xylose, OP and SCB hydrolysates was 79.1, 76.5, and 75.2%, respectively. The results obtained are of substantial value and pave the way for development of sustainable SA biomanufacturing in an integrated biorefinery.

## Highlights

• Xylose-rich hydrolysate a sustainable feedstock for SA production by *A. succinogenes*.
• SA titre and yield on pure xylose were 36.7 g/L and 0.27 g/g respectively.
• SA titres and yield of 28–34 g/L and 0.27 g/g achieved from SCB and OP hydrolysates.
• The recovery yield of SA on pure xylose, SCB and OP hydrolysates was > 75%.

## Graphical abstract

**Figure:**
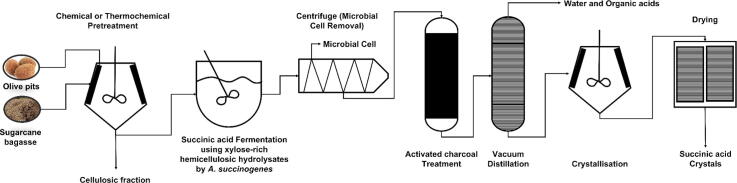

## 1 Introduction

Even today, the major fraction of fuels and chemicals are derived from the non-renewable fossil fuels. Current fossil-based chemical technologies suffer from non-sustainability, volatility in oil prices, negative environmental impacts and have led to resurgence of alternative routes. As a result of it, there is a paradigm shift from petrochemical to bio-based route which is sustainable and carbon–neutral (Vivek et al., 2021). The waste streams rich in fermentable carbon such as agricultural residues, industrial effluents, food and bakery wastes have become valuable resources in this era of circular bio/economy. The bioprocesses offer plethora of opportunities and have the potential to replace hydrocarbon-based production with carbohydrate economy using circular biorefining approach. The concept focuses on recycle, reuse and manufacture with cascading use of biologicals resources from various waste and side streams in a systematic manner, alleviating environmental concern and resulting in a low carbon economy (Zero Waste Scotland, 2017; Leong et al., 2021). Lignocellulosic biomass (LCB) is, one of the most widely available renewable carbons on the planet, inexpensive and rich in structural polysaccharides, cellulose, and hemicellulose. Majority of the work in literature have made use of cellulosic sugars for fermentative production of fuels and chemicals with limited research on hemicellulosic fraction rich in xylose (∼90%). Since majority of microbes lacks metabolic routes for utilization of pentose sugar, the hemicellulosic portion is overlooked. In the last two decades, various native and genetically engineered pentose assimilatory microorganisms have been identified and constructed that could valorise the fermentable sugars present in hemicellulosic fraction into an array of chemical products. The efficient conversion of pentose sugars will be essential for augmenting the economic viability and profitability of the LCB-based biorefineries (Kwak et al., 2019, Prabhu et al., 2020, Narisetty et al., 2021).

According to US Department of Energy, succinic acid (SA) is recognised as one of the 12 important platform chemicals attainable from biomass. SA is a natural C4-dicarboxylic acid intermediate of the tricarboxylic or citric acid cycle and has the potential to serve as a precursor for a variety of products, including commodity chemicals (and may be used to replace maleic anhydride), medicines, feed additives, green solvents, and biodegradable polyesters (Li et al., 2021, Prabhu et al., 2020). The biological production of SA has received great deal of attention in the last two decades. The bioproduction of SA has several advantages such as high fermentation efficiency, ability to use crude sources of fermentable carbon, renewability of substrates, biodegradability of substrates, intermediates, and products (Chen et al., 2021). The market of bio-based SA was estimated at $175.7 million in 2017 and has been forecasted to reach $900 million by 2026 with CAGR of 20%. The major global players involved in microbial succinic acid production are BioAmber, Myriant, Reverdia and Succinity with a total annual production of 76.6–86.6 kiloton SA (Li et al., 2021). However, the titers obtained from the biological process is not sufficient to accommodate global demand, hence major supply to the global market is through petrochemical route (Gao et al., 2016). Further, the high cost of SA production from biological route ($2.86–3.00/kg) in comparison to fossil-based production ($2.40–2.60/kg) is another challenge which stem out from costly feedstocks (pure sugars) and expensive product recovery. The production cost could be diminished with utilization of cost-effective renewable feedstocks and efficient downstream processing. The microbial SA production has been investigated, albeit to lesser extent using pure and crude xylose. Representative investigations to date have been focussed on SA manufacturing using pure and cellulosic/starch-based glucose (Dessie et al., 2018a, Li et al., 2021, Pateraki et al., 2016).

In the light of the above information, the current study was aimed to develop a bioprocess for manufacturing of SA using xylose-rich hemicellulosic hydrolysates derived from sugarcane bagasse (SCB) and olive pits (OP) by *A. succinogenes*. Initially, the optimal pure and crude xylose concentrations were evaluated in the shake flask experiments followed by validations in bench-top bioreactors. Further to improve the SA titers, fed-batch mode of fermentation was carried out using pure and crude substrates derived from SCB and OP hydrolysates. The fermented broth obtained from the fed-batch fermentation was subjected to downstream processing. The vacuum concentration combined with direct crystallisation by acidification approach was adapted for separation of SA under optimised temperature and operation conditions.

## 2 Materials and methods

### 2.1 Chemicals

All the chemicals used in this study were of reagent grade and procured from Sigma-Aldrich (St. Louis, MO, USA) and Acros Organics (New Jersey, USA).

### 2.2 Olive pits (OP) and sugarcane bagasse (SCB) hydrolysates

Crushed olive pits (OP) were subjected to dilute acid pretreatment (2%v/v H_2_SO_4_) with solid loading of 50% w/v at 121 °C for 30 mins in an autoclave. After the treatment, the slurry was brought to room temperature and the solid residue was filtered to separate the liquid fraction consisting of hemicellulosic sugars. The liquid fraction was termed as OP hydrolysate. The xylose rich SCB hydrolysate obtained by thermochemical pretreatment was provided by our industrial partner Nova Pangaea Technologies (https://www.novapangaea.com), Redcar, UK. The OP and SCB hydrolysates were concentrated by subjecting the liquid to vacuum distillation (Rotavapor, BUCHI UK Ltd), carried out at 100 mbar and 80 °C, overnight. The xylose concentration of ∼ 400 g/L was quantified in the concentrated hydrolysates.

### 2.3 Microorganism, culture maintenance and seed inoculum preparation

The strain *Actinobacillus succinogenes* DSM 22,257 was obtained from DSMZ, Leibniz Institute DSMZ-German Collection of Microorganisms and Cultures. The bacterial strain was maintained on TSA (Tryptic Soya Agar) agar plates, containing (g/L) 17, pancreatic digest of casein; 3, soy peptone; 2.5, glucose; 5, NaCl; 2.5, KH_2_PO_4_; and 18, agar. Stock cultures were revived using the supplier's procedure and the glycerol (20% w/v) stocks were stored at −80 °C. The seed culture (pre-inoculum) was prepared by inoculating a single bacterial colony from a freshly sub-cultured plate into 250 mL Erlenmeyer flask containing 50 mL of sterile Tryptic soy broth. After the inoculation, the flask was incubated for 16 h at 37 °C with an agitation speed of 150 rpm in a rotary incubator shaker .

### 2.4 Shake flask fermentation

Initial SA fermentation experiments to optimize the concentrations of pure xylose, OP, and SCB hemicellulosic hydrolysates (10 – 60 g/L xylose) were carried out in 250 mL Erlenmeyer flasks with 50 mL working volume. The fermentation media composition other than the carbon sources was as follows (g/L), 5, yeast extract; 0.3, Na_2_HPO_4_; 1.4, NaH_2_PO_4_; 1, NaCl; 1.5, K_2_HPO_4_; 0.2, MgCl_2_·2H_2_O; 0.2, CaCl_2_·2H_2_O The xylose concentration was adjusted as per the requirement of the experiment. Sterile MgCO_3_ (20 g/L) was supplemented to the fermentation media as source of CO₂ and as buffering agent to control the pH. The sterile fermentation media was inoculated using 10% v/v pre-inoculum prepared as per section 2.3. After inoculation, the flasks were incubated in the rotary shaker incubator at constant temperature and agitation of 37 °C and 150 rpm, respectively. Due to the biofilm formation, dark coloration of hemicellulosic hydrolysates and presence of MgCO_3_ into the fermentation media, optical density measurements were hard to perform, therefore, the values obtained may not be the true representation of bacterial cell growth during the fermentation (Salvachúa et al., 2016a, 2016b).

### 2.5 Bioreactor cultivation

Further to optimization studies in the shake flask experiments, the parameters were validated in the 2.5 L bench-top bioreactors with 1 L working volume. The SA fermentations were carried out in both batch and fed-batch mode in an autoclavable Electrolab bioreactor Fermac 360 stirrer unit (Electrolab biotech Ltd, Tewkesbury, United Kingdom). The pH, temperature and agitation speed were controlled at 7.0, 37 °C and 250 rpm without any aeration, respectively. The pH was maintained with an automatic addition of mixture containing 2.5 M NaOH and 2.5 M NaHCO_3_. The sodium bicarbonate salt can act as buffering agent as well as source of CO_2_.

### 2.6 Downstream processing

The optimization of temperature and agitation rate for downstream processing was performed using a synthetic solution consisting of 50 g/L SA. The SA fermentation broth consists of bacterial cells, insoluble and soluble macromolecules, and various byproducts. To separate and purify SA from the fermentation broth, the direct crystallisation method was adapted. The bacterial cells were separated by centrifugation for 10 min at 8000 rpm. After the centrifugation, the supernatant was separated from the bacterial pellet and treated with 2% w/v activated carbon for 2 h to decolourise the broth and remove residual protein impurities. In the direct crystallisation method, the pH of the aqueous broth was adjusted to 2.0 by the addition of 35% (v/v) HCl. Then, decolourised broth was subjected to vacuum distillation (Rotavapor, BUCHI UK Ltd) at 60 °C to remove carboxylic acids like formic and acetic acid and to concentrate the SA, followed by crystallisation at 4 °C with an agitation rate of 3000 rpm for 5 h. After crystallisation, the slurry was filtered with Whatman no 1 filter paper, and the crystals were collected by centrifugation at 8000 rpm and 4 °C for 10 min followed by drying of crystals at 70 °C for 24 h.

### 2.7 Analytical methods

Samples were withdrawn at regular intervals during the shake flask and bioreactor experiments to analyse for xylose, SA, ethanol, lactic acid (LA), acetic acid (AA), and formic acid (FA) concentrations using high performance liquid chromatography (HPLC) system. The samples were centrifuged at 10,000 rpm for 10 mins to remove the microbial cells and other suspended solids and further, the supernatant was filtered through a 0.22 μm nylon membrane (Sartorius, Germany). The filtered samples were eluted through Rezex ROA-Organic Acid H + (Phenomenex, USA) column, connected with Refractive Index Detector (RID) for sugars and Diode Array Detector (DAD) for organic acids. The mobile phase was 5.0 mM H_2_SO_4,_ and flow rate were 0.4 and 0.6 mL/min for sugars and acids, respectively. All the experiments were carried out in triplicates except bioreactor run which were performed in duplicates and the standard deviation observed was not>10%.

## 3 Results and discussion

### 3.1 Effect of pure xylose concentration on SA production

Glucose is the most preferred substrate for biological production of various chemicals and fuels. Xylose is the most abundant pentose and second major sugar after glucose in LCB hydrolysate. Due to lack of commercially viable microorganisms to utilise xylose, it is overlooked or considered as inferior fermentable sugar for decades which limits the economics of LCB-based biorefineries (Prabhu et al., 2020, Narisetty et al., 2021). For cost-effective biosynthesis of SA, there is a need for robust and efficient microorganisms that could utilize myriad of low cost and renewable feedstocks. *A. succinogenes*, a native SA producer, is a gram negative, capnophilic and facultative anaerobic strain with an ability to assimilate a wide range of carbon sources (Pateraki et al., 2016, Dessie et al., 2018a). The bacterium is a top performing cell factory for industrial SA bioproduction from diverse feedstocks and could indigenously metabolize xylose into SA. *A. succinogenes* has an incomplete/partial TCA cycle and make use of reductive branch to synthesize SA. The first step towards biosynthesis is carboxylation of C3 metabolites [Phosphoenolpyruvate (PEP) + CO_2_ + ADP → Oxaloacetate (OAA) + ATP] which is mediated through PEP carboxykinase. This reaction serves as branching point between C3 and C4 pathways. The OAA formed is converted to SA via malate and fumarate with reactions catalysed by malate dehydrogenase, fumarase and fumarate dehydrogenase (Dessie et al., 2018a, Dessie et al., 2018b, Yang et al., 2020).

**Fig. 1:**
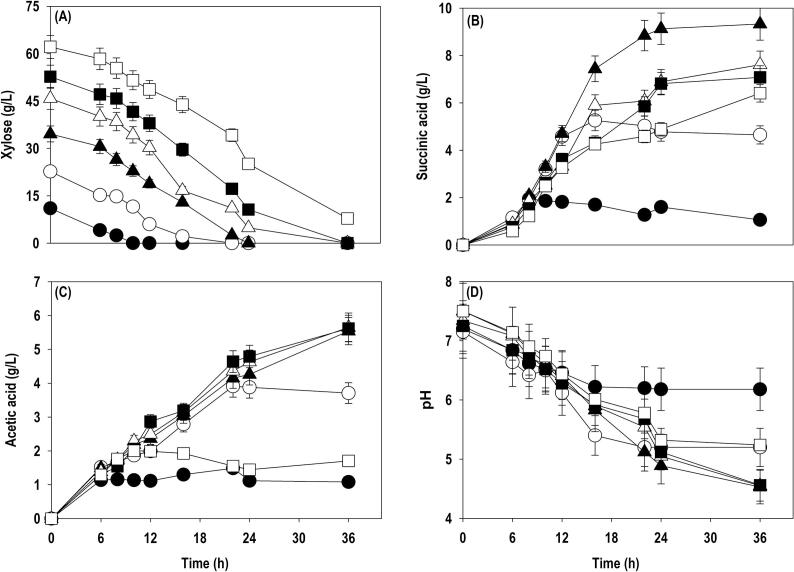
Shake flask cultivation of *A. succinogenes* at different levels of xylose: (A) xylose; (B) SA; (C) AA; (D) pH. Symbols: closed circle (10 g/L); open circle (20 g/L); closed triangle (30 g/L); open triangle (40 g/L); closed square (50 g/L); open square (60 g/L).

In the current study, we investigated *A. succinogenes* DSM 22,257 strain for xylose-based SA production*. A. succinogenes* was cultured in shake flasks using the media composition as described in section 2.4 with different initial xylose concentrations (10, 20, 30, 40, 50 and 60 g/L) to examine the impact of xylose on SA accumulation and determine optimal as well as inhibitory level. In case of bacterial fermentations, pH has been found an important parameter, especially during organic acid production. MgCO_3_ has been found most effective pH regulator among CaCO_3_, Na_2_CO_3_, NaOH, NH_4_OH, NaHCO_3_, Mg(OH)_2_, and Ca(OH)_2_ for SA production by *A. succinogenes* (Li et al., 2011, Liu et al., 2008, Pateraki et al., 2016). Therefore, the culture medium was supplemented with MgCO_3_ at 20 g/L to provide CO_2_ and Mg^2+^ ions which acts as cofactor for PEP carboxykinase catalysing carboxylation reaction. Fig. 1 shows the time course profiles for xylose uptake, SA production, AA accumulation and pH. Xylose was completely utilized with initial substrate levels of 10–50 g/L at 10, 22, 24 and 36 h, respectively, while at 60 g/L, only 87.4% of xylose was assimilated and a residual xylose concentration of 7.8 g/L was observed even after 36 h. There was a continuous improvement in SA titer with an increase in xylose concentration till 30 g/L and beyond, a decline in SA accumulation was noticed. The maximal SA titers obtained at 10, 20, 30, 40, 50 and 60 g/L xylose were 1.9, 5.3, 9.3, 7.6, 7.1 and 6.4 g/L, respectively. Similar trend was observed with SA yield and the highest SA yield of 0.27 g/g was recorded at 30 g/L xylose. Acetic acid (AA) was obtained as main by-product, 1.1, 3.7, 5.5, 5.6, 5.6 and 2.0 g/L was accumulated during cultivation on 10, 20, 30, 40, 50 and 60 g/L, xylose respectively. SA fermentations are accompanied by drop in pH which is also contributed through production of organic acids in the form of main product and byproducts. We also observed continuous decrease in pH during the course of fermentation. Beyond 20 g/L xylose, pH dipped below 5.0 which is not favourable for cell growth and SA production, as bacteria including *A. succinogenes* fermentation are quite sensitive to pH fluctuations and require near neutral pH values for optimal performance (Pateraki et al., 2016). The substrate inhibition effect appeared at 60 g/L xylose where low performance could be attributed due to the combined effect of high xylose levels and drop in pH.

### 3.2 Shake flask cultivation of *A. Succinogenes* on SCB and OP hydrolysates

The adoption of low-cost substrates is one of the strategies to curb the production cost of biological SA production. Huge amount of waste byproducts from LCB hydrolysis are rich in xylose with lesser concentrations of other sugars such as glucose, arabinose etc and can serve as cheap carbon source for sustainable bioproduction and SCB and OP are two such feedstocks. Sugarcane is a major sugar producing crop in Brazil, India, China, and other nations. SCB is major waste stream from sugar industries and largest agricultural residue with annual global production of 540 million metric tons (Chen et al., 2021, Konde et al., 2021). Olive oil is one of the most important foods in Mediterranean countries and olive cultivation and oil industries generates different waste streams, comprised of lignocellulosic materials which generally have no feed or other industrial applications and OP is one of them. In 2020 ∼ 16 million metric tons of olives were produced in about 5 million hectares of olive cultivation, majorly in Spain, Italy, Greece, Portugal, France and other European countries, and the OP or the endocarp constitutes 18–22% weight of the olive fruit, which is equivalent to ∼ 3.5 million tons (Valvez et al., 2021, Pantziaros et al., 2021). Currently, SCB and OP are incinerated to satisfy own energy demand of industries. This amount of waste presents a big opportunity for sustainable biorefineries. Further, SCB and OP are non-edible materials, so their use as feedstocks for refineries will have no impact on food chains for humans. Therefore, intensive research is needed to valorise these waste streams.

**Fig. 2:**
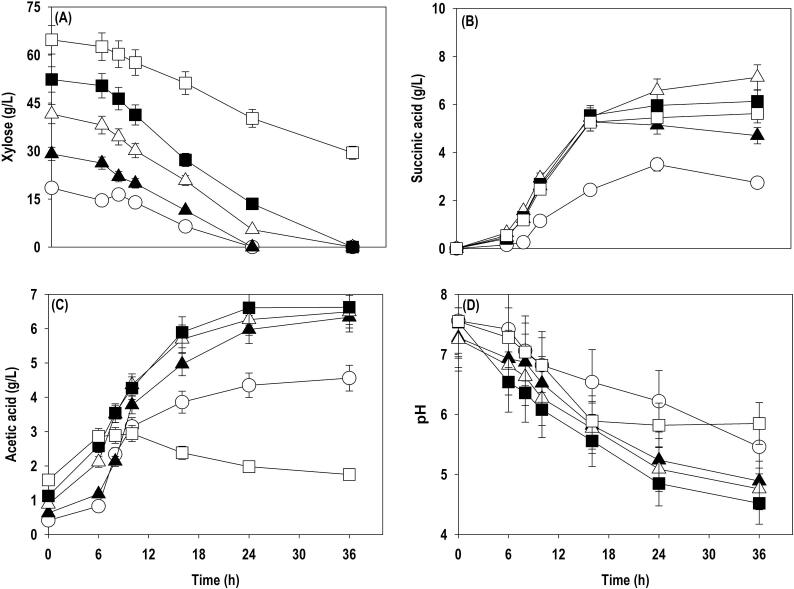
Batch cultivation of *A. succinogenes* in shake flask at different levels of xylose in SCB hydrolysate: (A) xylose; (B) SA; (C) AA; (D) pH. Symbols: open circle (20 g/L); closed triangle (30 g/L); open triangle (40 g/L); closed square (50 g/L); open square (60 g/L).

**Fig. 3:**
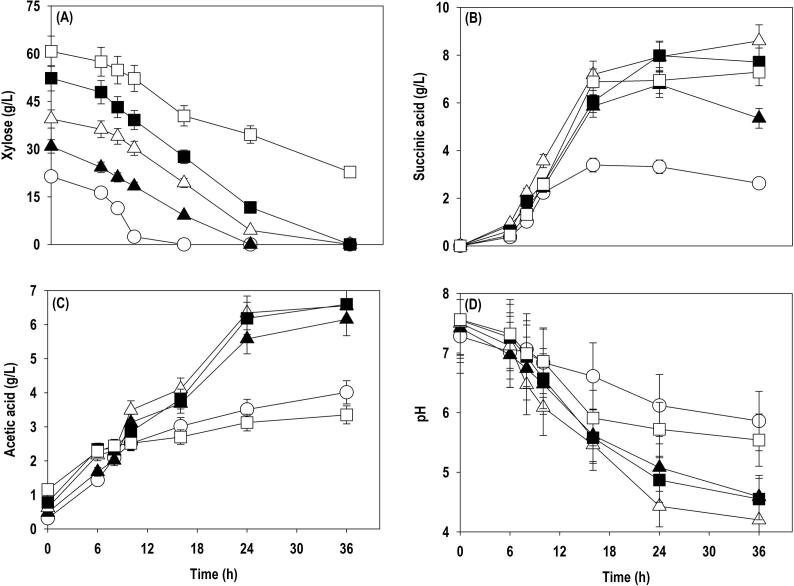
Time course profiles of residual xylose, pH, SA, and AA production by *A. succinogenes* in shake flask at different levels of xylose in OP hydrolysate: (A) xylose; (B) SA; (C) AA; (D) pH. Symbols: open circle (20 g/L); closed triangle (30 g/L); open triangle (40 g/L); closed square (50 g/L); open square (60 g/L).

After pure xylose, xylose rich hydrolysate obtained from SCB, and OP were employed for SA production. The concentrated hemicellulosic hydrolysates were diluted appropriately to maintain the desired concentration. Fig. 2, Fig. 3 represents the time series fermentation profile of xylose consumption, metabolite production (SA &AA) and pH using SCB and OP hydrolysates, respectively. The results obtained with both the hydrolysates were comparable with pure xylose. All the supplied xylose (20–50 g/L) was completely metabolized within 24–36 h. However, at 60 g/L xylose, a significant fraction (37–45%) remains unutilized and residual xylose at 36 h in case of SCB and OP hydrolysate was 29.5 and 22.8 g/L, respectively. In both the cases, the highest SA accumulation was observed at 40 g/L xylose with SA titer of 7.1 and 8.6 g/L and conversion yields of 0.17 and 0.22 g/g using SCB and OP hydrolysate, respectively. Beyond 40 g/L, SA titer and yields were dropped. All the fermentations were accompanied with AA accumulation and continuous reduction in pH and trend similar to pure xylose was observed. Despite the presence of fermentation inhibitors such as furfural, HMF, AA, phenolic compounds etc, the strain performed well on the hydrolysates. AA was present from the beginning and continuously accumulated during the fermentation, but we did not notice any inhibition, especially at 10–50 g/L as performance of the strain was similar in term of xylose assimilation and SA production on pure xylose, SCB and OP hydrolysates.

There are few studies where hemicellulosic hydrolysate from SCB has been used for microbial production of SA, but we did not come across any report making use of OP for SA production. Borges and Pereira, (2011) used *A. succinogenes* for SA production using pure as well as xylose-rich SCB hemicellulosic hydrolysate. They optimized the culture medium and SA accumulated using optimized medium composition supplemented with pure xylose and xylose-rich hydrolysate were 14.2 and 22.5 g/L with yield of 0.64 and 0.43 g/g, respectively. Like *A. succinogenes*, *Basfia succiniciproducens* is a facultative anaerobic bacterium with natural ability to produce SA from a variety of carbon sources. Salvachúa et al., (2016b) employed this bacterium for SA production from pure xylose, xylose-rich (∼70%) mock and real hydrolysate from corn stover. Mock hydrolysate contained four sugars (xylose, glucose, galactose, and arabinose), acetate, furfural and HMF while mock sugars consisted of these four sugars but without inhibitors. The batch fermentation in bioreactor with pure xylose, xylose-rich mock and real hydrolysate, each at 60 g/L, resulted in SA titers of 28.2, 25.9 and 30.6 g/L at 72 h. AA, LA and FA were obtained as main byproducts in all the fermentations. All these results are in agreement with our observation of comparable performance with pure xylose and hemicellulosic hydrolysates in terms of SA production.

### 3.3 Batch cultivation of *A. Succinogenes* in a bioreactor

**Fig. 4:**
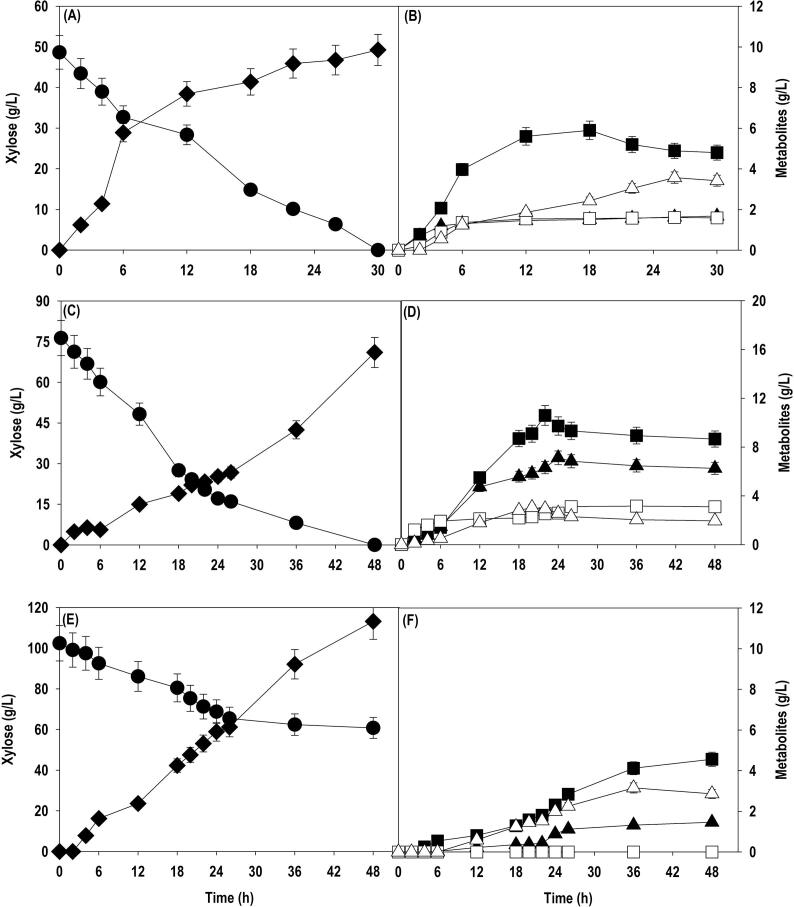
Batch culture of *A. succinogenes* in bioreactor at different concentration of pure xylose: (A)& (B) 50 g/L; (C)& (D) 75 g/L; (E)& (F) 100 g/L. Symbols: closed circle (xylose); closed diamond (SA); closed square (AA); closed triangle (FA); open square (LA); closed triangle (ethanol).

After shake cultivation, SA fermentations were performed in 2.5 L bench-top bioreactor in batch mode with different initial xylose concentrations: 50, 75 and 100 g/L. The pH was controlled using mixture of 2.5 M NaOH and 2.5 M NaHCO_3_. The mixture not only acted as pH regulator but also provided CO_2_. The time course profiles of xylose consumption and metabolite production [SA, AA, formic acid (FA), lactic acid (LA) and ethanol] are shown in Fig. 4. Like various reports on glucose utilization, rapid utilization of xylose was observed when the bioreactor was supplemented with 50 and 75 g/L xylose. The initial xylose level of 48.6 g/L was exhausted in 30 h leading to SA accumulation of 9.9 g/L with conversion yield of 0.20 g/g. AA (4.8 g/L), LA (1.6 g/L), FA (1.7 g/L) and ethanol (3.4 g/L) were obtained as byproducts. In case of 75 g/L, xylose uptake rate was faster and ∼ 50 g/L xylose was consumed in initial 18 h, however, the xylose assimilation was not translated into SA instead it was diverted towards byproduct formation. In fact, the amount of AA produced during 12–26 h was more than the main product SA and after 26 h, a significant enhancement in SA titer was observed. The fermentation time was prolonged with complete utilization of xylose in 48 h resulting in total SA production of 18.9 g/L with conversion yield of 0.25 g/g. AA (8.7 g/L) was obtained as major byproduct followed by FA (6.3 g/L), LA (3.1 g/L) and ethanol (2.0 g/L). The inhibitory effects were observed with initial xylose level of 100 g/L. The fermentation progressed slowly and ∼ 30 g/L xylose was consumed in first 24 h followed by assimilation of about only 8 g/L xylose in next 24 h. As a result of it, ∼61 g/L xylose remain unconsumed even after 48 h. Similar trend was noticed with SA production and titer of 11.3 g/L was obtained at the end of fermentation with AA (4.6 g/L), FA (1.5 g/L) and ethanol (2.9 g/L) as byproducts.The biosynthesis of SA is strongly dependent on availability of NADH and ATP produced by Glycolytic pathway and byproduct formation contributes towards generation of these cofactors (Yang et al., 2020). As a result of it, SA production is accompanied with byproducts formation. The biosynthesis of AA is accompanied with ATP formation and is one of the main sources of energy after glycolytic pathway under oxygen limited conditions. This becomes more important in case of xylose where ATP yields are lower than glucose (Pateraki et al., 2016, Salvachúa et al., 2016a). Similarly, FA metabolism provides CO_2_ and reducing power in the form of NADH (Yang et al., 2020).

The literature reports on xylose-based SA are scarce. We came across one report (Salvachúa et al., 2016a) where impact of initial xylose concentration (40–100 g/L) on substrate consumption and SA production by *A. succinogenes* was evaluated. Similar to our results, 40–60 g/L was completely depleted within 20–40 h and inhibitory effects were visible at 80 and 100 g/L. The bacteria population metabolized 95% of 80 g/L in 72 h and xylose utilization further sowed down at 100 g/L, ∼60% of xylose was assimilated after 72 h of fermentation. The cell growth and SA productivity were declined at initial xylose concentrations of 80 – 100 g/L. In another report, Chen et al., (2011) made use of *A. succinogenes* NJ113 for synthesis of SA from a variety of carbon sources including xylose with biotin-supplemented yeast cell hydrolysate as the nitrogen source. The batch fermentation was started with 70 g/L xylose and only 53.7% xylose was assimilated in 42 h leading to SA accumulation of 22.6 g/L indicating substrate inhibition at higher xylose levels. The sugar mixture containing 31.4 g/L of glucose, 25.5 g/L of xylose, and 14.2 g/L of arabinose yielded 45.1 g/L SA under similar condition with 97.3% utilization of sugars. carried out continuous anaerobic SA fermentation using xylose as substrate by *A. succinogenes*. The fermentation was performed at different dilution rates and the highest SA titer (30.8 g/L) was recorded at 0.05 h^−1^ with AA (5.5 g/L) and FA (2.3 g/L) as main by-products. Besides *A. succinogenes*, other microbial cell factories have been examined for SA production from xylose. Andersson et al., (2007) investigated the ability of recombinant *E. coli* AFP184 for manufacturing SA from glucose, fructose, xylose, and their mixtures. The dual phase fermentation consisting of an aerobic and anaerobic phase was employed and SA was accumulated during anaerobic phase which was maintained by continuously sparging CO_2_. When using xylose (100 g/L) as sole carbon source, the strain generated ∼ 25 g/L SA with conversion yield of 0.50 g/g during anaerobic phase. All these findings including this study, suggests that xylose could be a promising substrate for the microbial production of SA.

### 3.4 Fed-batch cultivation of *A. Succinogenes*

**Fig. 5:**
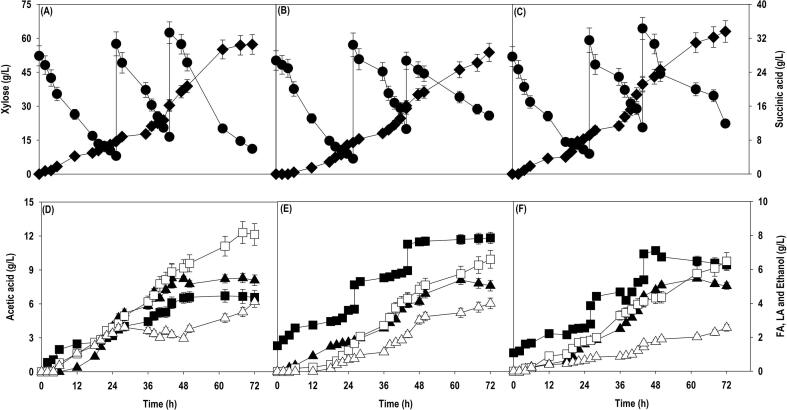
Fed-batch cultivation of *A. succinogenes* in bioreactor on: (A)& (D) pure xylose; (B)& (E) SCB hydrolysate; (C)& (F) OP hydrolysate. Symbols: closed circle (xylose); closed diamond (SA); closed square (AA); closed triangle (FA); open square (LA); closed triangle (ethanol).

Fed-batch is preferred mode of operation with controlled addition of substrate to eliminate substrate inhibition and enable high titer of end-product which is highly desired at an industrial scale. After the batch cultivation, SA fermentation using pure xylose, SCB and OP hydrolysates were performed in a fed-batch mode with initial xylose level of 50 g/L. A feed of concentrated (400 g/L) xylose solution was used for intermittent replenishing of xylose in the culture medium, when the residual levels were dropped to 20 g/L or less during the fermentation. Fig. 5 shows the variation in xylose consumption, and metabolite accumulation using pure xylose, SCB and OP hydrolysate, respectively. The results achieved with pure xylose were used as a baseline against which SA production from SCB and OP hydrolysate could be compared. The results obtained with SCB and OP hydrolysates compare well with the baseline xylose fermentations. About 80% of initial xylose concentration was consumed within 24 h, thereafter the culture was fed two times at 26 and 44 h. The fermentation can be divided into three phases: phase I (0 – 26 h); phase II (26 – 44 h); phase III (44 – 72 h). In case of pure xylose, the substrate assimilation rate was almost unaffected in phase II and III whereas with SBC and OP hydrolysate, substantial reduction in xylose uptake rate was observed in phase III. As a result of it, 22–25 g/L xylose was left unconsumed at 72 h. The SA production in phase I was in range of 7–10 g/L with further improvement in phase II and III. The significant difference in biosynthesis of SA was noticed in phase III where maximum product accumulation was observed with pure xylose in comparison to SCB and OP hydrolysate which is concomitant with xylose uptake rate. The final SA titer achieved with pure xylose, SCB and OP hydrolysate were 36.7, 33.6 and 28.7 g/L, respectively, with same conversion yield of 0.27 g/g. *A. succinogenes* metabolized the xylose efficiently whether it is in the pure or crude form, explaining the efficiency of the strain to utilize LCB feedstocks for production of SA. Like shake flask and batch bioreactor fermentation, AA, FA, LA, and ethanol were obtained as main byproducts with concentration < 10 g/L. The SA accumulation could be significantly improved if the carbon loss in the form of byproducts could be channelized towards SA formation.

Besides being abundant, xylose is readily released from LCB with acid treatment without any requirement of enzymes. Many independent and parallel efforts have been made to manufacture SA from crude renewable sources via biological route. However, there are handful of studies making use of fermentable sugars from SCB for SA production and few have them have already been explained above. Xi et al., (2013) carried out the batch fermentation in bioreactor using non-detoxified hemicellulosic hydrolysate from SCB containing 22.4 g/L xylose, 3.6 g/L glucose and 3.9 g/L arabinose. The SA titer and yield achieved at the end of fermentation (24 h) were 23.7 g/L and 0.79 g/g with complete consumption of all sugars. Surprisingly, the non-detoxified hydrolysate yielded better results than detoxified one. In another study, Chen et al., (2016) extracted SCB sugars using alkali pretreatment followed by enzymatic hydrolysis which yielded a reducing sugar concentration of 55 g/L with glucose and xylose in ratio of 3:1. The fed-batch culture of *A. succinogenes* using cellulosic plus hemicellulosic hydrolysate resulted in 70.8 g/L SA with conversion yield of 0.82 g/g at 50 h. Corona-González et al., (2016) performed acid (HCl and H_2_SO_4_) and enzymatic hydrolysis of *Agave tequilana* Weber bagasse. The acid hydrolysis yielded mainly xylose while enzymatic one released glucose as major sugar and fermentation of sugars from hydrolysates by *A. succinogenes* yielded SA titre in range of 4.0 – 6.0 g/L with substantial amount of AA and FA. They also immobilized *A. succinogenes* in agar and conducted repeated batch fermentation using sugars from enzymatic hydrolysate. The total amount of SA accumulated after five cycles in 40 h was 33.6 g/L using 87.2 g/L total monosaccharides. Two reports on SA production using xylose enriched hydrolysate from corn stover by *A. succinogenes* were published by NREL, USA. In one of the reports (Salvachúa et al. 2016a), SA fermentation was performed in a batch mode using pure xylose, mock and real hydrolysates. The maximum SA concentration obtained with pure xylose (80 g/L) was 48.0 g/L in 72 h with 95% xylose utilization. The study reported detoxification of furfural to furfuryl alcohol via reduction by *A. succinogenes* and growth analysis presented that acetate as the main inhibitor in the hydrolysate. The corn stover was deacetylated using mild alkaline wash, before dilute acid (H_2_SO_4_) pretreatment. The SA titer of 42.8 g/L was achieved using the deacetylated DAP hydrolysate with yield and productivity of 0.74 g/g and 1.27 g/L. h, respectively. The purpose of deacetylation was to eliminate acetic acid accumulation during the dilute acid pretreatment and remove alkali soluble lignin. In another study from same research group (Bradfield et al., 2015), a continuous cultivation was conducted with immobilized *A. succinogenes* using pure xylose and non-detoxified, xylose-rich corn stover deacetylated hydrolysate from dilute acid pretreatment. They also found that the concentrations of furfural and HMF were reduced and subsequently decreased to zero during the course of fermentation. The SA titre, yield, productivity with pure xylose and hydrolysates were 32.5 g/L, 0.77 g/g, 2.64 g/L. h and 39.6 g/L, 0.78 g/g and 1.77 g/L. h, respectively. However, titres obtained were lower in comparison to batch cultures in their previous work.

The SA titers obtained in current study compare well with most of the literature, however, yield and productivity are lower. One of the possible reasons could be the limited availability of carbon dioxide which is a co-substrate in biosynthesis of SA by *A. succinogenes*. The SA production by *A. succinogenes* is highly dependent on CO_2_ levels as the dissolved CO_2_ regulates the carbon metabolic flux and stimulates the activity of PEP carboxykinase, the key enzyme for SA production via reductive cycle. The direct CO_2_ supply through sparging could redirect the flux of xylose to SA biosynthesis and minimizes the by-product formation (Dessie et al., 2018b, Yang et al., 2020). In the present study, MgCO_3_ was added to fermentation medium and for bioreactor cultivation, pH was controlled using a mixture of NaOH and NaHCO_3_ to provide CO_2_. We believe that CO_2_ provided through these carbonates and indirect CO_2_ donors, was not sufficient to maintain high dissolved levels. Further process optimization and cultivations in anaerobic conditions with continuous purging of CO_2_ could help in improving the production parameters. We achieved SA yield of 0.27 g/g on xylose which is much lower than the maximum theoretical yield of 1.12 g/g. Some carbon can be accounted for cell mass and byproduct formation, the remaining difference is likely due to undetected/unknown metabolites, which may have been synthesized through alternative metabolic routes. We believe that this can be an extensive study and more work is required to decode this.

### 3.5 SA recovery from simulated solution and fermented broth

Downstream processing is considered as the bottleneck in the commercialization of fermentative production of chemicals as the separation and purification of desired product from the fermented broth is challenging task and strongly impacts the process economics (Kurzrock and Weuster-Botz, 2010).

#### 3.5.1 Optimization of temperature and agitation rate

Crystallisation is a well-known process for the separation of organic acids and sugar alcohols. Temperature, pH, and agitation speed are the important parameters affecting the outcome of the crystallization process (Thuy et al., 2017). Various literature reports have mentioned that pH 2.0 is optimal for the maximum recovery of SA from the aqueous phase or fermented broth. Organic acids exist in undissociated and dissociated/salt forms and their ratio depend on pH value. There is significant difference between solubility of undissociated and dissociated forms and at low pH values, major fraction of organic acid is present in undissociated form which facilitates product recovery. In all the experiments below, pH value of 2.0 was used for SA recovery through crystallization. In the process of crystallisation, nucleation and crystal growth are the two important steps, resulting in the partition of SA from the liquid to solid phase. Temperature trigger the nucleation and crystal growth by affecting the solubility and the supersaturation behaviour of the SA in the aqueous phase (Xiao et al., 2020). Similarly, agitation rate influences the mass transfer efficiency from liquid to solid phase and enhances the crystal growth.

In order to investigate the effect of temperature and agitation on the SA recovery, a simulated solution (pH – 2.0) consisting of 50 g/L SA and 10 g/L AA was used. The influence of temperature on recovery yield of SA in temperature range of 4 – 8 °C at 1000 rpm was investigated. The process was carried out for 5 h. At 8 °C, a little SA recovery of 2.4% was obtained and further drop in temperature caused significant improvement in the product recovery to 4.3, 15.2, 31.1 and 35.0% at 7, 6, 5 and 4 °C, respectively. Further, the impact of agitation rate from 1000 to 5000 rpm on the SA recovery yield was examined at pH and temperature of 2.0 and 4 °C, respectively. At 1000 rpm, 35.7% SA recovery was observed, we found that as agitation rate was increased, 45.3% at 2000 rpm, and maximum SA recovery of 55% was recorded at 3000 rpm, SA recovery was continuously enhanced till 3000 rpm and further increase to 4000 and 5000 rpm caused marginal drop in the recovery to 52.7 and 51.3%, respectively. It was also observed that the AA remained in solution form indicating that SA can be selectively crystalized.

#### 3.5.2 Influence of initial SA concentration on recovery

Further to the operation conditions like temperature, and agitation rate, the concentration of SA is also an important parameter to be considered for crystallisation, and recovery yield (Li et al., 2010). Henceforth, the effect of initial SA concentration (50 – 150 g/L) on the recovery yield was then investigated using the optimal parameters identified above (pH: 2.0; temperature: 4 °C; agitation speed: 3000 rpm). The highest recovery yield of 74.8% was achieved at initial SA concentration of 150 g/L, and an average of 72.5 % was observed between 75 and 150 g/L SA. Similar recovery yield (72.4%) was also obtained by Law et al., (2019), using a simulated solution consisting of 110 g/L SA through direct crystallisation approach.

#### 3.5.3 Separation and purification of SA from the fermented broth through crystallisation

After optimizing the temperature, agitation speed and initial SA level with simulated solution, SA was recovered from fermented broth accumulated on pure xylose, SCB and OP hydrolysate. For this, 100 mL of fermented broth was subjected to initial centrifugation and decolorization as described in section 2.6, after the treatment, the supernatant was filtered through Whatman No. 1 filter paper for removing the AC. The clear fermented broth obtained after the AC treatment was concentrated and further crystallized at 4 °C and 3000 rpm for 5 h. The maximum SA recovery yield of 79.1, 75.2 and 76.5 % was obtained with pure xylose, SCB and OP hydrolysates, respectively. An average recovery yield of 70% with direct crystallisation after acidification (Li et al., 2010) and 89% with direct crystallisation after resin treatment (Lin et al., 2010) was observed. Till date the maximum SA recovery yield of 79% has been achieved by Alexandri et al., (2019), using simulated solutions of xylose based fermentation media components. Our results shows that the one step direct crystallisation after acidification is an effective technique for the separation and purification of the SA from the aqueous solutions and fermented broth without any need for additional unit operations unlike precipitation resulting in > 70% of recovery yield. Further investigations need be carried out to improve the yield in combination of other strategies like salting-out or aqueous two-phase extraction for commercial viability of the bioprocess.

## 4 Conclusion

Economics of the bioprocess majorly dependent on feedstock to produce biochemicals through fermentative route; therefore, use of cost-effective renewable sources are of significant interest for commercial viability of biorefineries. The current study investigated the SA manufacturing ability of *A. succinogenes* from SCB and OP, two major agro-industrial waste streams. The result shows that non-detoxified xylose-rich hemicellulosic fractions can be utilised for production of SA without any inhibition and demonstrates the towering potential of *A. succinogenes*. Furthermore, a product recovery > 75% gives stimulus for future work which will be directed at pathway/metabolic engineering and process optimization for improving the primary fermentation metrics (titer, yield, and productivity) for industrial implementation.

## CRediT authorship contribution statement

**Esther Oreoluwa Jokodola:** Conceptualization, Methodology. **Vivek Narisetty:** Conceptualization, Methodology. **Eulogio Castro:** Methodology. **Sumit Durgapal:** . **Frederic Coulon:** . **Raveendran Sindhu:** Methodology. **Parameswaran Binod:** Methodology. **J. Rajesh Banu:** . **Gopalakrishnan Kumar:** . **Vinod Kumar:** Supervision, Conceptualization, Data curation.

## Declaration of Competing Interest

The authors declare that they have no known competing financial interests or personal relationships that could have appeared to influence the work reported in this paper.
